# Next Generation Leaders Programme: A Multi-Methods Evaluation of a Leadership Development Programme for Biomedical Researchers

**DOI:** 10.2147/AMEP.S386961

**Published:** 2022-12-22

**Authors:** Harry Kingsley-Smith, Sarah Short, Koot Kotze, Oscar Lyons

**Affiliations:** 1 Department of Medical Education, Oxford University Hospitals NHS Foundation Trust, Oxford, Oxfordshire, UK; 2 Nuffield Department of Surgical Sciences, University of Oxford Medical Sciences Division, Oxford, Oxfordshire, UK; 3 Nuffield Department of Primary Care Health Sciences, University of Oxford, Oxford, Oxfordshire, UK

**Keywords:** leadership, graduate medical education, graduate education, research personnel

## Abstract

**Background:**

Biomedical scientists have become de facto leaders for their research teams. Theories of expert leadership suggest that the specialist knowledge and credibility these researcher-leaders bring to their roles can lead to improved performance. Formal leadership development for biomedical researchers remains uncommon, and it is unclear whether existing leadership development programmes achieve improved individual and organisational outcomes. Our study evaluates the effectiveness of a single centre leadership development programme for biomedical researchers using a mixed-methods approach.

**Methods:**

26 biomedical researchers participated in an 8 month single centre multidisciplinary leadership development programme. Participants completed prospective pre-test, retrospective then-test and traditional post-test self-assessments using the Primary Colours Questionnaire (PCQ) and Medical Leadership Competency Framework Self-Assessment Tool (MLCFQ). Pre–post pairs and then–post pairs were analysed for changes using Wilcoxon signed-rank tests and compared with a parallel mixed-methods evaluation organised by Kirkpatrick levels.

**Results:**

There were significant increases in 3/7 domains and 1/5 tasks of leadership in the PCQ, in both pre-post and then-post paired assessments. There were statistically significant but small increases in 2/7 domains of the MLCFQ. The mixed-methods data showed positive outcomes at all Kirkpatrick levels. Participants said the programme was relevant, interesting and well-organised, with 63% reporting increased confidence and motivation. Participants had a significant change in behaviour, spending 1–2 hours per week on group projects, which were successfully implemented locally. 42% of participants expected these projects to continue beyond the programme.

**Discussion:**

This study demonstrates a local leadership programme can have positive impact within a biomedical research centre despite time and financial constraints. We encourage future studies to utilise a mixed-methods approach to evaluating the impact of leadership development programmes.

## Introduction

Biomedical research teams have become larger and more multidisciplinary,^1^ Part of this reflects the multidisciplinary nature of clinical service delivery. Part can also be attributed to larger funding of research infrastructure. For example, in the UK, the National Institute for Health and Care Research (NIHR) has recently invested £790 million in Biomedical Research Centres to drive innovation in health research.^2^ As a result of both the need for innovative clinical research and the subsequent investments, biomedical scientists have become de facto leaders for their research teams. However, despite skills in applied research and knowledge translation, formal leadership training has yet to be established at most biomedical science institutions.^3^

The employment of accomplished researchers as leaders within their institutions is correlated with improved future university performance.^4^ Theories of Expert Leadership suggest that the specialist knowledge and credibility these researcher-leaders bring leads to improved performance,^4^ in line with the correlation between clinician-leaders and hospital performance.^5^ It is less clear whether leadership development in these organisations also leads to improved performance and outcomes.^6–8^ We were unable to find any systematic reviews of leadership development for biomedical researchers.

The present study aims to evaluate the effectiveness of a single centre leadership development programme for biomedical researchers using a mixed-methods approach.

## Materials and Methods

### Setting

This study evaluated the first cohort of the Next Generation Leaders Programme (NGLP), based at a single UK Biomedical Research Centre funded by the NIHR. The NGLP was developed from an existing local leadership development programme for practicing healthcare professionals. The programme design was based on a distributed leadership model, which has been widely used for leadership development in education and healthcare.^9^,^10^ The programme supports early career biomedical researchers to develop leadership capacity through leadership project work and workshops.^7^,^11^

Forty biomedical researchers applied for the programme and 26 were accepted. Acceptance was based on applicant availability for workshop dates and their purported desire to apply learning into practice (identified using two short application essays). Participants included those with both patient facing and non-clinical biomedical research roles, and were at career stages ranging from doctoral students to senior postdoctoral researchers (see Table 1).

**Table 1:** Participant Demographics. All Doctoral Students Had a Clinical Background. Dropout Was Most Significant Amongst Post-Doctoral Researchers and Research Managers. Clinical Background Was Defined as Having Had a Patient-Facing Role

	Started Programme n (%)	Completed Programme n (%)
**Gender**		
Female	18 (69%)	12 (63%)
Male	8 (31%)	7 (37%)
**Career Stage**		
Doctoral Student	6 (23%)	6 (32%)
Post-Doctoral Researcher	13 (50%)	8 (42%)
Senior Post-Doctoral Researcher	2 (8%)	2 (11%)
Research Manager	5 (19%)	3 (16%)
**Background**		
Clinical	14 (54%)	9 (47%)
Non-Clinical	12 (46%)	10 (53%)
**Total Participants**	**26 (100%)**	**19 (76%)**

**Notes**: Bold text illustrates the headings of participants demographics and the headings of each data column. Bold text also highlights the final row indicating the total number of participants at the start and end of the programme.

Group leadership projects formed the main foundation for leadership learning. Participants proposed projects during the launch session which the faculty then screened for alignment with organisational strategic goals, feasibility and complexity. Participants ranked project proposals and self-organised into multidisciplinary teams with between three and six members. They worked on their project throughout the programme, presenting progress at each of the workshops and presenting project outcomes at the final workshop.

Learning through the group projects was supported by seven half-day workshops delivered over eight months (October 2019 to May 2020). As a result of the Covid-19 pandemic the final two sessions were delivered remotely (see Supplementary Materials). Workshops consisted of interactive discussion from experienced biomedical research leaders (to inspire participants and humanise research leadership), structured group learning sessions (to introduce knowledge and skills of leadership) and facilitated group project work (to provide a vehicle for experiential learning).

### Evaluation

Impact was assessed across all four levels of Kirkpatrick’s framework for training programmes, as adapted for medical education research.^12^,^13^ Data was collected using multiple methods including pre- and post-programme self-assessment questionnaires, pre- and post-programme Brief Resilience Scales,^14^ workshop feedback surveys, free-text questions, systematic observations, and programme project outcomes (henceforth, “mixed-methods” data). Informed consent was obtained using a participant information sheet.

Two questionnaires were used to evaluate participants’ self-assessed change in leadership skills. Participants completed these questionnaires pre-and post-programme, with a retrospective then-test (“what were you like back then”) added to each item at the post-programme evaluation. The two questionnaires used were the Primary Colours Questionnaire (PCQ)^15^ and an adapted version of the Medical Leadership Competency Framework Self-Assessment Tool (MLCFQ) (see Supplementary Materials), both of which have been assessed by experts for face validity and were developed from established leadership theories.^16^ The Next Generation Leaders programme content and methods were mapped to the tasks and domains of leadership identified within the PCQ (see Supplementary Materials). In the PCQ, participants rated their leadership ability on 12 items using a 10-point Likert scale anchored to 1=very poor and 10=excellent. In the MLCFQ, participants rated themselves on 56 behaviours grouped into the 7 MLCFQ self-assessment domains, using a 7-point Likert scale anchored to 1=strongly disagree and 7=strongly agree. The combined score for each of the seven MLCFQ domains was scaled to 1–7 for ease of interpretation.

### Data Analysis

Changes in PCQ and MLCFQ scores for each of the 12 PCQ items and each of the seven MLCFQ domains were compared using Wilcoxon signed-rank tests for pre–post/post-test pairs and then-post/post-test pairs. Alpha was set at the 0.05 level and the Bonferroni correction for multiple comparisons was applied in each set of tests. Participants who did not complete the course due to COVID were excluded from analysis.

Workshop feedback scores and post-programme-only scores were analysed using descriptive statistics.

Free-text comments were analysed by thematic analysis, using Kirkpatrick’s levels^17^ as a sensitising framework.

## Results

### Programme Completion

19 of the 26 participants (73%) completed the programme (see Table 1 above). The majority of participants who dropped out were of a clinical background. Participants who dropped out cited personal reasons or increased pressures relating to COVID-19.

### Leadership Self-Assessments

**Figure 1:**
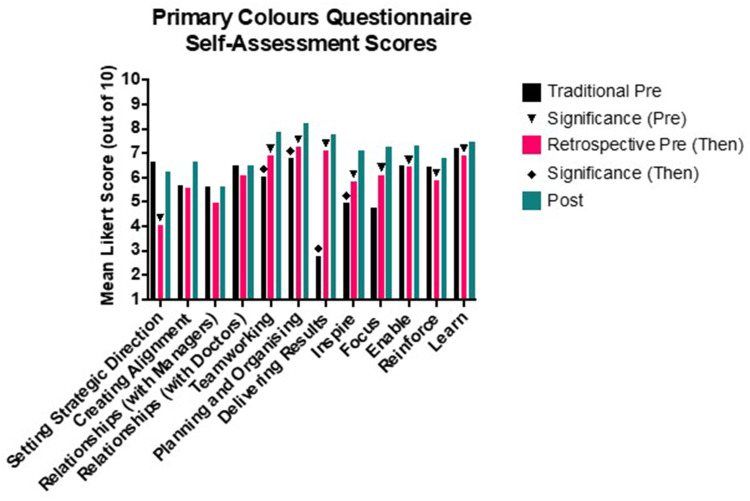
Mean Primary Colours Questionnaire (PCQ) Scores before and after the programme. Symbols indicate statistical significance. Traditional Pre refers to Ratings on a traditional pre-course questionnaire. Retrospective pre (Then) refers to ratings participants gave of themselves as they recall they had been at the start of the course. Post-test refers to ratings participants’ ratings of themselves at the end of the course. The significance symbols are differentiated for the traditional (Pre) and retrospective (Then) test for clarity.

In the PCQ there were significant increases in three of the seven domains of the PCQ (Teamworking, Planning & Organising, Delivering Results) and one of the five tasks (Inspire) using both prospective and retrospective self-assessments (see Figure 1). In addition, there were significant changes in “Setting Strategic Direction” and all of the remaining Tasks (Focus, Enable, Reinforce, Learn) when analysing retrospective (then-post) pairs alone.

While there was a statistically significant increase in two of the MLCFQ domains, identified using retrospective then-post pairs. However, these increases were small, particularly in comparison to those identified in the PCQ which are explored below.

### Resilience

There were widely varying shifts in Brief Resilience Scale (BRS) scores across the course, with mixed increases and decreases for different individuals. There was no significant change in the BRS overall. The Supplementary Questionnaire did contain a neutrally phrased item related to resilience: “Has the programme affected your resilience?”. In this item, 53% stated their resilience had increased and no participant reported a decrease.

### Mixed-Methods Data

The programme demonstrated positive impact on all four of Kirkpatrick’s levels, as shown in Table 2. Free-text comments were broadly positive and described participants believing they had successfully developed their leadership skills and ability to think analytically and critically about leadership theory. One participant felt the “programme has made me realise how little empirical evidence there is for a lot of management training… a lot of it is quite unscientific.” The programme also led to a change in behaviour as participants spent a significant amount of time working on their group projects each week. Several participants stated that they expected to continue work on their group projects beyond the formal conclusion of the programme. Many also reported an improvement in team dynamics within their normal research groups, which they attributed to the application of skills they had learnt during the programme. Additionally, one participant successfully obtained grant funding during the later stage of the programme, for which they attributed credit to the NGLP for improving their confidence.

**Table 2:** Mixed-Methods Data, Grouped by Kirkpatrick Level

	Programme Outcomes
**Level One: Reaction** *Participants’ views on the learning experience, its organisation, presentation, content, teaching methods and quality of instruction.*	Participants rated each session from 1=poor to 10=excellent. Mean ratings from a subset of workshops* were 8.05 (Relevance), 7.79 (Interest), 7.03 (Fun), and 7.94 (Organisation).
**Level Two: Attitudes/Knowledge** *Changes in the attitudes or perceptions among participant groups towards teaching and learning; acquisition of concepts; acquisition of thinking/problem-solving, psychomotor and social skills.*	In free-text comments participants reported better understanding of leadership and the development of their confidence and communication skills: “The biggest impact of the programme was on my confidence, assertiveness and communication skills” “This programme has provided my with the practical skills to take forward in my career in terms of leadership”. “I feel I understand the theory behind being a good leader”. These comments were supported by questionnaire data, where 63% of participants reported increased confidence and motivation.
**Level Three: Behaviour** *Transfer of learning to the workplace or willingness of learners to apply new knowledge and skills.*	On average, participants spent 1–2 hours per week on their projects in their free time 42% expected their project work to continue beyond the programme 26% reported increased job satisfaction, however, one participant (5%) notably reported a decrease.
**Level Four: Results** *Changes in the organisation, attributable to the educational program; improvement in student or resident learning/performance as a* *direct result of the educational intervention.*	Project impact included: The redesign of the hospital’s clinical trials website with a new-patient friendly layout. A biomedicine outreach careers day, with positive feedback from attendees and researchers. Implementation of occupational health posters Designing of a web-based toolkit for breastfeeding and milk expression, hosted on a departmental web page. Additionally: 53% of participants reported increased resilience, though this was not reflected in BRS scores One participant partly attributed their success in a grant application to the programme Several participants noted improvements in their research team dynamics, which they attributed to application of learning from the programme

**Notes**: *Please note technological difficulties meant only the third, fourth and sixth workshops were rated by participants. Bold text denotes each column’s heading as well as the Kirkpatrick level in each row. Italicised text summarises the aspects of each Kirkpatrick level.

**Abbreviations**: NGLP, Next Generation Leaders Programme. BRS, Brief Resilience Scale.

### Changes Recommended by Participants

Suggested changes varied across participant responses and in some cases directly conflicted with other participants’ responses. Many participants suggested that no changes were needed and “the program went well in all aspects”. There were a number of areas in which several participants suggested changes, particularly relating to the project work which formed the foundation of the programme. These changes suggested all related to learning methods rather than content. We have noted these areas in Table 3.

**Table 3:** Participant Feedback on the Learning Methods Used Within the Programme. Bold Text Denotes Each Column’s Heading. Italicised Text Denotes a Summary of Participant Feedback About a Learning Method

Summary Points of Participant Feedback About Learning Methods	Explanation of Participant Feedback
Projects were time consuming but important for learning	Participants were surprised at the amount of time they ended up spending on their team projects. They also noted that the projects were of significant use in terms of their learning. One participant said they were “…hesitant at first but think [the project] worked really well.” Some participants stated that they had not realised there would be project work involved in the programme, despite this being clearly stated on all of the programme materials and application instructions.
Projects should be arranged ahead of the programme start	There was a general feeling that participants would have liked more clear direction in terms of project topics from the start, and would have preferred for the project topics to have come from the organisational or course leaders. “I would have preferred to be assigned to something challenging that really needed doing (Ie projects set by the course leaders)”.
Project mentors might have been helpful	Participants suggested that it would have been helpful to have mentors for their projects, who would be able to help them navigate the complexity of a change project: “I think mentoring would also be a helpful addition”.
Participants would like more scientific appraisal of some of the teaching material	Participants noted that while they appreciated the practical focus of the workshops, they would have liked to have seen “slightly more scientific appraisal” of the teaching material.
Workshops could be made longer to make space for more interaction	Participants suggested that longer workshops would enable more interaction and engagement with material, alongside more small group exercises and time for group project work: “it would have been helpful to have longer sessions”.
Speakers were generally excellent, particularly in the second half of the programme (after a speaker briefing sheet was introduced)	Participants noted variation in the quality of invited speakers, with most speakers being “very insightful and interesting”, and some speakers being less engaging: “Some speakers were more helpful than others as role models and people to learn from”.

## Discussion

Participants who completed the programme generally reported that the workshops were relevant, interesting, enjoyable and well organised. There were statistically significant improvements in participant leadership skills using the PCQ and the MLCFQ instruments, although the changes in the MLCFQ were too small to be meaningful. Through group project evaluations and individual comments, there appeared to be meaningful organisation impact from the programme (Kirkpatrick Level 4). The mixed-methods data suggest that the programme was successful in improving participants’ leadership skills.

We were interested to note that there was little overall change in the MLCFQ outcomes. This contrasts with a previous methodological study where there was broad concordance between the PCQ and MLCFQ outcomes.^15^ The COVID-19 pandemic occurred partway through the programme and resulted in a quarter of participants needing to leave the programme. It also resulted in changes to clinical practice for many participants, programme workshops being moved online, and in many cases reduced the personal contact between research team members. As the MLCFQ is based more heavily on behavioural self-assessment questions than the PCQ, these changes in practice may have impacted the MLCFQ more than the PCQ, which is based on self-assessed ability across the domains and tasks of leadership.

There was marked variability in the BRS resilience scores for participants in the programme, which contrasted with self-reported improved resilience by most participants. The self-reported impact on resilience question was likely more vulnerable to social desirability bias than the validated BRS.^14^ It is also clear that the Covid-19 pandemic introduced a considerable stressor for participants, given that several participants who left the programme cited it as their reason for dropping out. It will nonetheless be worth incorporating the 6-item validated BRS into future healthcare leadership education programmes to investigate the impact of leadership development programmes on resilience in the absence of such a confounder as COVID-19, including, if possible, an appropriate control-group to control for environmental changes.

We noted that the retrospective then-post question pairs showed a greater number of significant item increases than the prospective pre-post pairs. This was most evident in the PCQ tasks of leadership, where retrospectively all five tasks showed a significant improvement, whereas only “Inspire” showed a significant prospective increase. It is possible the retrospective pairs were either more sensitive at detecting change or liable to overinflating change due to bias, as discussed in a previous paper^15^ and by experts in retrospective assessments.^18^

Of those who completed the programme, it should be noted one participant reported that they did not enjoy the programme. They felt the initial speakers were

mostly talking about their own experiences …. [which was] not very applicable to most people in the room.

As a group, participants found variation in the quality of invited speakers. A speaker brief was incorporated in the second half of the programme in response to early participant feedback. Invited speakers were asked to inspire and humanise leadership and use their experiences to highlight teachable moments relevant to scenarios participants could, in the future, find themselves in. After this briefing was added, participants’ feedback regarding speakers became more positive in nature, with speakers being highlighted as excellent in the post programme free-text comments. In future programmes, it would be worthwhile collecting data relating to other barriers that participants may have experienced with respect to learning and transfer of learning, so that these barriers can be addressed.

In line with best practices in healthcare leadership evaluation,^7^,^19^ we incorporated multiple quantitative and qualitative methods in this evaluation, assessed longitudinal outcomes, and tailored the methods to the aims of the programme, including organisational impact (Kirkpatrick level 4). While the MLCFQ and PCQ instruments have been developed from theory and assessed by experts for face validity, they still rely on participant self-assessments and are thus vulnerable to bias, including “response-shift” bias,^20^ bias from an implicit theory of change and error justification bias.^15^,^18^ We employed both prospective and retrospective self-assessments in an attempt to partially mitigate these risks of bias. There remains no established objective method of evaluation of a healthcare or biomedical leadership development programme. In future programmes that incorporate team leadership projects, it would be worth explicitly collecting data regarding how participants behave in these projects and how they apply their learning.

In conclusion, this study demonstrates a leadership programme can have some positive impact within a biomedical research centre despite limited time and financial funding. Given the relative lack of rigorous evaluation within the field of leadership development programmes, we hope this programme will encourage others to not only invest in educating biomedical researchers in leadership, but also to contribute to the growing literature by evaluating their leadership development efforts. Where possible, evaluations of biomedical research leadership development programmes should be tailored to the specific programme aims, incorporate multiple qualitative and quantitative methods, and include longitudinal and objective components.
